# Nursing; Its Principles and Practice, for Hospital and Private Use

**Published:** 1906-09

**Authors:** 


**Nursing; its Principles and Practice, for Hospital and Private Use.** By **Isabel Hampton Robb,** Graduate of the New York Training School for Nurses, Bellevue Hospital; late Superintendent of Nurses and Principal of the Training School for Nurses, Johns Hopkins Hospital, Baltimore. Third edition. revised and enlarged. 12mo. 565 pages. Cleveland: E. C. Koeckert. 1906.

The author of this book, formerly Miss Isabel Hampton, is now the wife of the distinguished Cleveland surgeon, Hunter Robb, and is a woman as prominent in social, philanthropic and educational circles as she formerly was in her profession.

The book has been revised to meet the requirements of the three years' course of study which is being adopted of necessity by training-schools in New York and voluntarily in many other states.

In the opening chapter is presented a suggestive outline for a three years' course of instruction, which, to the mind of the reviewer is superior to the one recently presented by the State Board of Nurse Examiners. Following this, the second and subsequent chapters take up those subjects which belong to the elementary instruction of the pupil nurse, as the hygiene of the ward and sick-room, ventilation, light, disposal of excreta, and the like.

The subject of sterilisation and disinfection follows next, some instruction in bacteriology being advised as preparatory to a proper understanding of the subject. The pupil nurse next takes up general nursing, beds and bed making, the care of a patient in bed, convalescence, baths of various kinds, in fact all the procedures a nurse should be acquainted with before being set to work on a particular case. Then follow chapters on nursing in general, medical diseases, surgical and gynecological nursing, nursing in infectious diseases, nursing of children, obstetrical nursing, and nursing in certain special diseases, as affections of the eye, ear, etc. The whole field of nursing is covered thoroughly and well.

The work is not intended to instruct the nurse fully in the art of nursing. No one volume can suffice for that with the present standard of education. But as a guide for the superintendent and nurse instructors, and as a textbook for pupil nurses it is eminently satisfactory, up-to-date and thorough.

An excellent reference list for supplementary reading and study is given in the opening chapter. M. J. F.

